# A Critical Tryptophan and Ca^2+^ in Activation and Catalysis of TPPI, the Enzyme Deficient in Classic Late-Infantile Neuronal Ceroid Lipofuscinosis

**DOI:** 10.1371/journal.pone.0011929

**Published:** 2010-08-03

**Authors:** Salomon Kuizon, Kathleen DiMaiuta, Marius Walus, Edmund C. Jenkins, Marisol Kuizon, Elizabeth Kida, Adam A. Golabek, Daniel O. Espinoza, Raju K. Pullarkat, Mohammed A. Junaid

**Affiliations:** 1 Department of Developmental Biochemistry, New York State Institute for Basic Research in Developmental Disabilities, Staten Island, New York, United States of America; 2 Department of Developmental Neurobiology, New York State Institute for Basic Research in Developmental Disabilities, Staten Island, New York, United States of America; 3 Department of Molecular Biology, New York State Institute for Basic Research in Developmental Disabilities, Staten Island, New York, United States of America; Case Western Reserve University, United States of America

## Abstract

**Background:**

Tripeptidyl aminopeptidase I (TPPI) is a crucial lysosomal enzyme that is deficient in the fatal neurodegenerative disorder called classic late-infantile neuronal ceroid lipofuscinosis (LINCL). It is involved in the catabolism of proteins in the lysosomes. Recent X-ray crystallographic studies have provided insights into the structural/functional aspects of TPPI catalysis, and indicated presence of an octahedrally coordinated Ca^2+^.

**Methodology:**

Purified precursor and mature TPPI were used to study inhibition by NBS and EDTA using biochemical and immunological approaches. Site-directed mutagenesis with confocal imaging technique identified a critical W residue in TPPI activity, and the processing of precursor into mature enzyme.

**Principal Findings:**

NBS is a potent inhibitor of the purified TPPI. In mammalian TPPI, W542 is critical for tripeptidyl peptidase activity as well as autocatalysis. Transfection studies have indicated that mutants of the TPPI that harbor residues other than W at position 542 have delayed processing, and are retained in the ER rather than transported to lysosomes. EDTA inhibits the autocatalytic processing of the precursor TPPI.

**Conclusions/Significance:**

We propose that W542 and Ca^2+^ are critical for maintaining the proper tertiary structure of the precursor proprotein as well as the mature TPPI. Additionally, Ca^2+^ is necessary for the autocatalytic processing of the precursor protein into the mature TPPI. We have identified NBS as a potent TPPI inhibitor, which led in delineating a critical role for W542 residue. Studies with such compounds will prove valuable in identifying the critical residues in the TPPI catalysis and its structure-function analysis.

## Introduction

The mammalian tripeptidyl-peptidase I (TPPI, EC 3.4.14.9) is a crucial endopeptidase involved in cleaving off tripeptides during lysosomal protein catabolism [1]–[3]. The importance of functionally active TPPI is evident from a debilitating neurodegenerative disorder called the classic late-infantile neuronal ceroid lipofuscinosis (LINCL, OMIM 204500), also known as neuronal ceroid lipofuscinosis 2 or Jansky-Bielschowsky disease [4], [5]. LINCL is a familial, fatal autosomal-recessive disorder afflicting children, which is characterized by progressive retinal and neuronal degeneration [6]. LINCL, whose symptoms appear between 2 and 11 years of age in affected individuals, is caused by mutations in the gene encoding TPPI, denoted at *TPP1*. The resultant defective protein was first identified through a proteomics approach [7]. Initially recognized as a protein similar to bacterial carboxypeptidase, the gene product was quickly identified as TPPI [8], [9]. Histologically, the disorder is characterized by the accumulation of characteristic curvilinear autofluorescent storage bodies, ceroid lipofuscin, primarily in the brain, but also in other tissues. The severity and the onset of clinical symptoms of LINCL depend upon the type of mutation, some of which cause the complete loss of functional activity, while others result in altered proteins that retain partial peptidase activity. The latter class of mutations results in a protracted phenotype with delayed onset of symptoms and delayed mortality [10], [11]. At present, there are 68 different disease-causing mutations in the *TPP1* gene among different individuals listed in the NCL mutational database (http://www.ucl.ac.uk/ncl/).

Currently, no efficacious treatment for LINCL is available; treatment is mainly restricted to symptomatic relief. The course of the disease ultimately leads to early mortality. Prevention through genetic counseling is the only means for avoiding the occurrence of LINCL, and several prenatal and carrier status testing assays are available [12]–[17]. A number of promising therapeutic approaches are also under investigation in the mouse models of LINCL [18]–[20]. Structure-function studies of TPPI are necessary to identify the molecular mechanisms associated with various mutations occurring in LINCL to develop proper strategies for rational therapeutic intervention. Expression of TPPI is augmented in breast, esophageal and colorectal cancers suggesting its likely involvement in cancer progression and metastasis [21]–[23]. In metastasis, it is generally believed that due to the action of various proteases, the basal lamina between epithelium and stroma separates, resulting in detachment of metastatic cells. Increased TPPI activity was also noticed in other neurological disorders probably as a secondary consequence [24], [25].

*TPP1*, is localized on chromosome 11p15, and the mRNA transcribed encodes a ∼69-kDa inactive zymogen precursor. The precursor TPPI is glycosylated on four of the putative N-glycosylation sites, with a final addition of a terminal mannose-6-phosphorylation modification that assists in lysosomal sorting [26]–[28]. After targeting into lysosomes, because of the acidic environment, the TPPI is auto-catalytically processed into the ∼48-kDa mature enzyme wherein it participates in protein catabolism in conjunction with other proteases by facilitating the removal of tripeptides [29]. It has been shown recently that the prosegment of TPPI acts as an inhibitor as well as a molecular chaperon of the mature enzyme, helping deliver the required peptidase activity into the the lysosome [30]. In TPPI, the glycosylation of N residues has been indispensable for the biological function. The mutation N286S in an LINCL patient was shown to cause the lack of a crucial glycosylation that abrogated the peptidase activity [31]. *In vitro* site-directed mutagenesis studies modifying the N286 residue have provided conclusive evidence that glycosylation at this residue is critical for the proper targeting of TPPI into the lysosome [27].

The crystal structures of both native and deglycosylated TPPI were contrived recently, which has helped unravel the molecular function of several mutations in the *TPP1* gene [32], [33]. TPPI is a mammalian representative of the S53-type family of serine proteases, and it is characterized by the presence of a bacterial subtilisin-like fold, a catalytic triad comprising S475-E272-D360 residues and an octahedrally coordinated Ca^2+^. Prokaryotic examples of S53-type serine proteases include sedolisin, sedolisin B, and kumamolisin [34]. Both the mammalian and prokaryotic members of S53-type proteases share common features, such as catalytic residues, pH optima, and substrate specificities. Chemical modification studies along with site-directed mutagenesis have provided unequivocal proof of the involvement of S475 in the active site of TPP1. The sequence of active-site residues is conserved in all members of the S53 family of proteases.

Substrate-specificity studies have shown that TPPI primarily cleaves tripeptides from unsubstituted amino termini in peptides and proteins. A substitution of the amino group on the P1 residue in a peptide completely abolishes the TPPI activity. Studies with combinatorial peptide libraries have indicated that substrates containing a hydrophobic residue at P1 and a positively charged residue at P3 were most favored [35]. In this aspect, TPPI appears to be a general peptidase involved in protein catabolism in concert with other lysosomal proteases. Apart from the primary tripeptidase activity, weaker endoproteolytic activity was also detected in TPPI [36]. Although not proved conclusively, the presence of this weaker endoproteolytic activity may be necessary for the autocatalytic processing of the precursor into the mature enzyme. Despite vigorous attempts by various groups, the natural substrates for TPPI have not been identified. *In vitro* TPPI cleaves a number of naturally occurring peptides such as mitochondrial ATP synthase subunit c, angiotensin II, substance P, β-amyloid, glucagons, and Bid, a Bcl2-interacting protein [36]–[38]. To date, in LINCL, accumulation of only subunit c has been demonstrated to be a component of autofluorescent ceroid lipofuscin [39]. While *in vitro* studies have shown subunit c to be a substrate, its accumulation has been reported in other neurological conditions; therefore, subunit c accumulation does not solely results from a lack of TPPI activity [40].

In out continuing efforts to delineate amino acid residues that are critical in TPPI catalysis, we have found that N-bromosuccinimide (NBS) is a potent inhibitor of purified TPPI. In this report, using biochemical and molecular biological approaches, we present evidence that W residue at position 542 is critical for TPPI activity. It is likely that NBS oxidized the indole group of this residue, which molecular models show, forms tertiary interactions with other residues in TPPI. Furthermore, we have shown that Ca^2+^ is essential for the autocatalytic processing of the precursor zymogen to active TPPI, but is not necessary for the tripeptidyl peptidase activity.

## Results

### TPPI activity is inhibited by *in vitro* incubation of NBS

Our initial studies were done with the apparently homogenous bovine brain TPPI that was purified as described earlier [37]. The purified TPPI was stored frozen at −20°C without any noticeable loss in activity; furthermore, during extended periods of storage at 4°C, no proteolytic fragments were observed upon SDS-PAGE separation followed by silver staining, indicating the lack of any self-endoproteolytic activity in the purified TPPI fractions. The purified TPPI is potently inhibited by freshly prepared NBS in a concentration-dependent manner (Table 1) under the acidic conditions of the assay. Total inactivation of peptidase activity was observed with a NBS concentration as low as 10 µM, whereas 6 µM NBS caused over 60% inhibition. The NBS inhibition of TPPI is also time-dependent, and maximum inhibition with any concentration of NBS is seen within 5 minutes incubation at 37°C (data not shown). NBS caused an irreversible-concentration dependent inhibition of the TPPI at all concentrations tested, suggesting that the inhibitor covalently modifies amino acid residue(s) in the enzyme. The TPPI activity was not recovered by dialyzing out NBS.

**Table 1:** Concentration-dependent inhibition of bovine brain TPPI by NBS.

NBS concentration (µM)	TPPI activity (% Inhibition)
1.56	9±1
3.12	24±1.4
6.25	66±2.2
12.5	97±3.4
25	100±2

TPPI was preincubated with the freshly prepared NBS solution at the indicated concentration at 37°C for 5 min. Following preincubation, the tetrapeptide substrate was added, and TPPI assay performed as described in Experimental Procedures. Values are represented as mean ± SD for three independent measurements.

NBS is known to oxidize W residues in proteins at acidic pH. While only W residues are modified at lower pH, free sulfhydryl groups from cysteine residues are preferentially modified at higher pH [41]. The reaction of sulfhydryl groups with NBS is reversible with dithiothreitol [42]. In mature human TPPI, there are five sulfhydryl groups from C residues, four of which are involved in disulfide bond formation, leaving only one free sulfhydryl group. There are no known missense mutations involving the free C residue, according to the NCL mutational database (http://www.ucl.ac.uk/ncl/), indicating that this residue is not critical for TPPI catalysis. Because NBS inhibition of TPPI was observed at an acidic pH of 3.5, and it was irreversible, we reasoned that one or more of the W rather than the C residues might be oxidized in the TPPI. Additionally, that would also implicate the significance of W residues in TPPI catalysis.

### Site-directed mutagenesis of TPPI to identify the critical W residue

**Figure 1:**
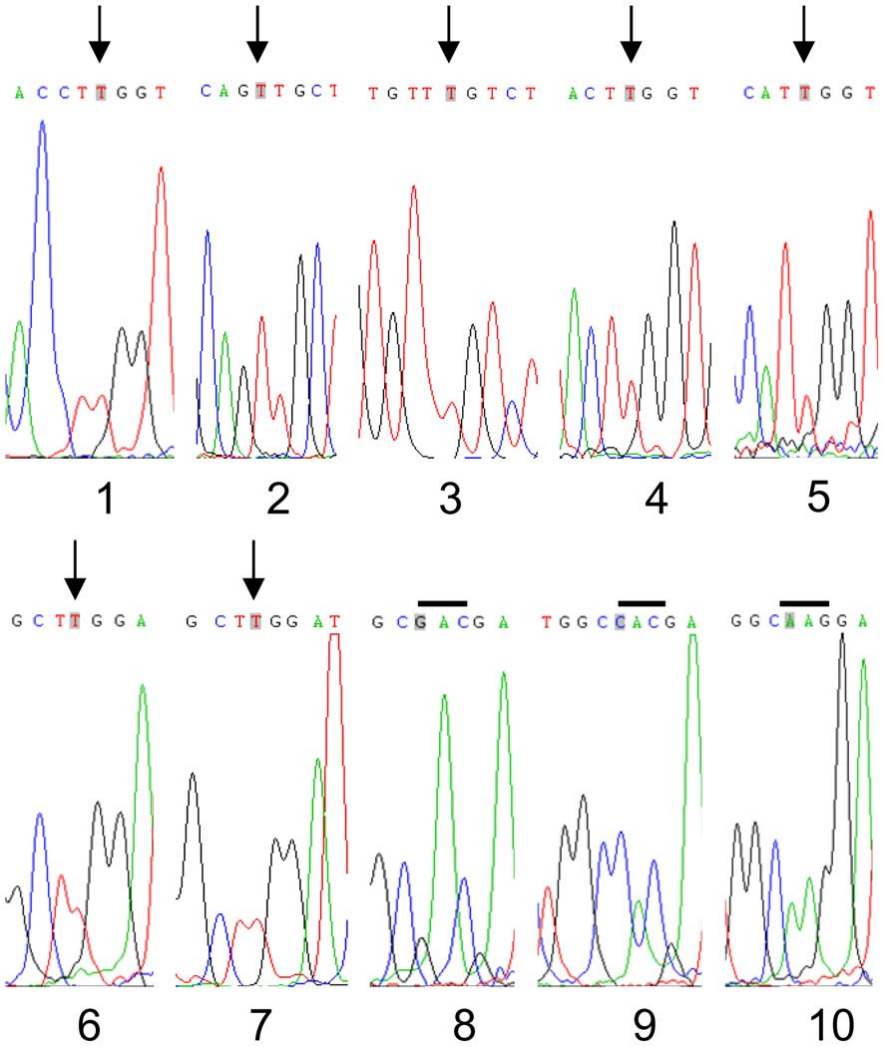
Site-directed mutagenesis of W residues in TPPI and DNA sequencing. W residues were numbered 1–7 starting from the amino-terminus. The corresponding amino acid positions in the mature TPPI are 1, W290; 2, W307; 3, W366; 4, W460; 5, W470; 6, W542; and 7, W548. Arrows indicate the nucleotide change from g→t. Sequence numbers 8–10 are W542D, H, and K mutants, respectively. The bar represents the codon replaced to change the W residue.

In human TPPI, there are altogether 10 W residues; three of these are removed by autocatalysis in the propeptide, leaving seven W residues in the mature TPPI. All the W residues in known mammalian TPPI that are reported in GenBank database are conserved (data not shown). In order to identify the specific W residues critical for TPPI catalysis, we have systematically replaced each of the W residues in human gene sequence with the neutral amino acid L, and evaluated the enzyme activity of each mutant. Because the inhibition of TPPI activity was seen with the purified enzyme, we modified W residues only in the sequence of the mature enzyme. Mutations were introduced by site-directed mutagenesis, using synthetic primer pairs encompassing the mutation site (Table 2). A single g nucleotide in the tgg codon for W was changed to t nucleotide in the ttg codon for L. The complete sequence of the TPPI insert in each purified plasmid was verified by direct dideoxy chain termination DNA sequencing in both forward and reverse directions with the primers described in “Experimental Procedures” (the partial sequence determined by DNA sequencing is shown in Figure 1).

**Table 2:** Primer sequences for introducing various W mutants in the TPPI sequence.

Primer	Nucleotide	Mutation	Primer sequence (5′ to 3′)
CLN2W1F	g869t	W290L	5′-gccaacatctccaccttggtctacagtagcc-3′
CLN2W1R	g869t_antisense		5′-ggctactgtagaccaaggtggagatgttggc-3′
CLN2W2F	g920t	W307L	5′-gagcccttcctgcagttgctcatgctgc-3′
CLN2W2R	g920t_antisense		5′-gcagcatgagcaactgcaggaagggctc-3′
CLN2W3F	g1097t	W366L	5′-gtggggccgggtgtttgtctgtctctgg-3′
CLN2W3R	g1097t_antisense		5′-ccagagacagacaaacacccggccccac-3′
CLN2W4F	g1379t	W460L	5′-ctgcactttctgatggctacttggtggtcagca-3′
CLN2W4R	g1379t_antisense		5′-tgctgaccaccaagtagccatcagaaagtgcag-3′
CLN2W5F	g1409t	W470L	5′-caacagagtgcccattccattggtgtccggaac-3′
CLN2W5R	g1409t_antisense		5′-gttccggacaccaatggaatgggcactctgttg-3′
CLN2W6F	g1625t	W542L	5′-ctgctctggtcctggcttggatcctgtaaca-3′
CLN2W6R	g1625t_antisense		5′-tgttacaggatccaagccaggaccagagcag-3′
CLN2W7F	g1643t	W548L	5′-gggatcctgtaacaggcttgggaacaccc-3′
CLN2W7R	g1643t_antisense		5′-gggtgttcccaagcctgttacaggatccc-3′
CLN2W6DF	g1625t	W542D	5′-ctgctctggtcctggcgacgatcctgtaaca-3′
CLN2W6DR	g1625t_antisense		5′-tgttacaggatcgtcgccaggaccagagcag-3′
CLN2W6KF	g1625t	W542K	5′-ctgctctggtcctggcaaggatcctgtaaca-3′
CLN2W6KR	g1625t_antisense		5′-tgttacaggatccttgccaggaccagagcag-3′
CLN2W6HF	g1625t	W542H	5′-ctgctctggtcctggccacgatcctgtaaca-3′
CLN2W6HR	g1625t_antisense		5′-tgttacaggatcgtggccaggaccagagcag-3′

Each of the primer pairs was used to introduce the indicated mutation in the TPPI wt cDNA cloned into the plasmid pcDNA 3.1, by site-directed mutagenesis. The entire plasmid was amplified by PCR and transfected into competent *E.coli* cells, and positive colonies were screened by DNA sequencing.

### TPPI activity in W mutants

**Figure 2:**
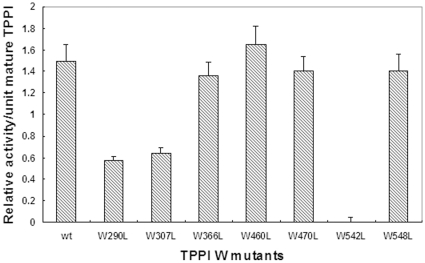
Specific activity of TPPI for various W mutants relative to mature TPPI. Freshly plated CHO cells at 70% confluency were transiently transfected with mutant plasmids using FugeneHD reagent. After 48 h, cells were pelleted by centrifugation at 800× g for 5 min, followed by homogenization in lysis buffer comprising 50 mM ammonium formate, pH 3.5, containing 0.15M NaCl and 0.1% Triton X-100. TPPI activity was measured as described in Experimental Procedures and related to the band intensity of mature TPPI by Western blot analysis.

To measure the activity of wild-type (wt) and mutant TPPI, confluent CHO cells were transiently transfected by either circular wt or each of the L-modified TPPI mutant plasmids. After 48 h, the cells were collected by brief centrifugation at 800× g, washed once with normal saline, and then homogenized in 50 mM ammonium formate buffer, pH 3.5, containing 0.15M NaCl and 0.1% Triton X-100. After centrifugation, the clear supernatant was stored at −20°C until used for TPPI activity and protein concentration measurements. Transient transfection of CHO cells by the wt and mutant TPPI resulted in a 2- to 3-fold higher peptidase activity over endogenous CHO cells activity (Table 3). The extract from CHO cells transfected with mutant plasmid W542L had only endogenous TPPI activity, indicating that this W residue is crucial for enzyme catalysis. All other CHO cell extracts transfected with other W mutants had between 2- and 3-fold higher TPPI activities, thereby suggesting that these W residues are not critical for TPPI activity. A complete lack of peptidase activity in the W542L mutant confirms our initial finding of total inhibition of purified bovine TPPI in the presence of NBS. To rule out the degree of transfection as a possible factor for the lack of TPPI activity, we also related the TPPI activity based upon the intensity of the band of mature protein by Western blot analysis (Figure 2). Again, the mutant W542L showed the complete loss of TPPI activity and the mature protein.

**Table 3:** TPPI activity in various W mutants.

Mutation	% TPPI activity
CHO cells	100±6
wt	310±8
W290L	260±10
W307L	205±9
W366L	310±11
W460L	355±10
W470L	325±13
W542L	100±5
W548L	255±8

CHO cells were transiently transfected with various W mutants created by site-directed mutagenesis. After 48 h, cell pellets were prepared by centrifugation at 800× g for 5 min, rinsed once with normal saline and extracts made in ammonium formate buffer, pH 3.5, containing 0.15M NaCl and 0.1% triton X-100. TPPI activity was measured as described in Experimental Procedures. Values are represented as mean ± SD for three transfections.

### Structural requirement of the residue at position 542

For the initial essential amino acid residue screening, we have reduced the electron donor capacity of the side chain at residue 542 by replacing it with a neutral L residue. This resulted in complete loss of TPPI activity. To identify structural requirements for position 542, we have replaced the W with other amino acids using the same site-directed mutagenesis strategy outlined earlier (Table 2). Three more mutant plasmids were constructed that possessed either D, H, or K residues at position 542 (Figure 1). The presence of acidic D residue or basic H and K residues also resulted in the substantial loss of TPPI peptidase activity. The inhibition, however, was not complete, and between 18 and 35% of the wt TPPI peptidase activity was recovered (Table 4), the highest being with the H mutant. These results suggest that the presence of an electron donor at position 542 is critical for the peptidase activity of TPPI.

**Table 4:** Structural requirements for the residue at position 542 for TPPI activity.

Residue at 542	% TPPI activity
W (wt)	300
D	118±1
H	135±2
K	129±2

CHO cells were transiently transfected with plasmids comprising various amino acids at position 542. After 48 h, cell pellets were prepared by centrifugation at 800× g for 5 min and rinsed with normal saline, and extracts were made in ammonium formate buffer, pH 3.5, containing 0.15M NaCl and 0.1% triton X-100. TPPI activity was measured as described in Experimental Procedures. Values are represented as mean ± SD for three transfections.

### Localization of mutant TPPI

**Figure 3:**
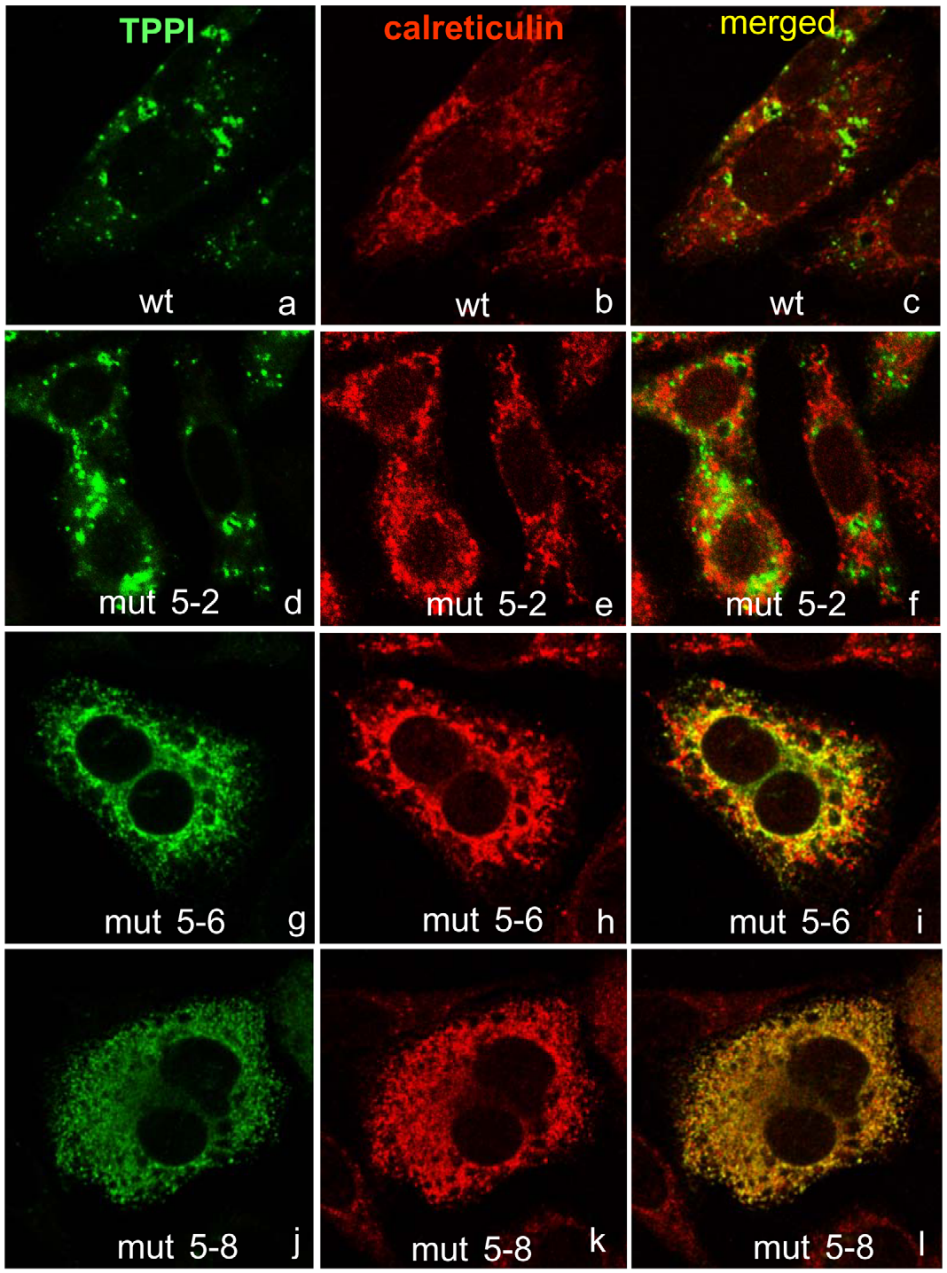
Localization of wt and mutant TPPI in CHO cells. Freshly plated CHO cells at 70% confluency were transiently transfected with mutant plasmids using FugeneHD reagent. After 24 h, cells were immunostained for TPPI (8C4, monoclonal) and calreticulin (rabbit polyclonal). Secondary antibodies labeled with Alexa Fluor 488 (green fluorescence visualizing TPPI) and Alexa Fluor 555 (red fluorescence visualizing calreticulin) were used. Upon merging, the yellow color indicates overlap of expressed proteins. Digital images were taken at a magnification of ×1000.

**Figure 4:**
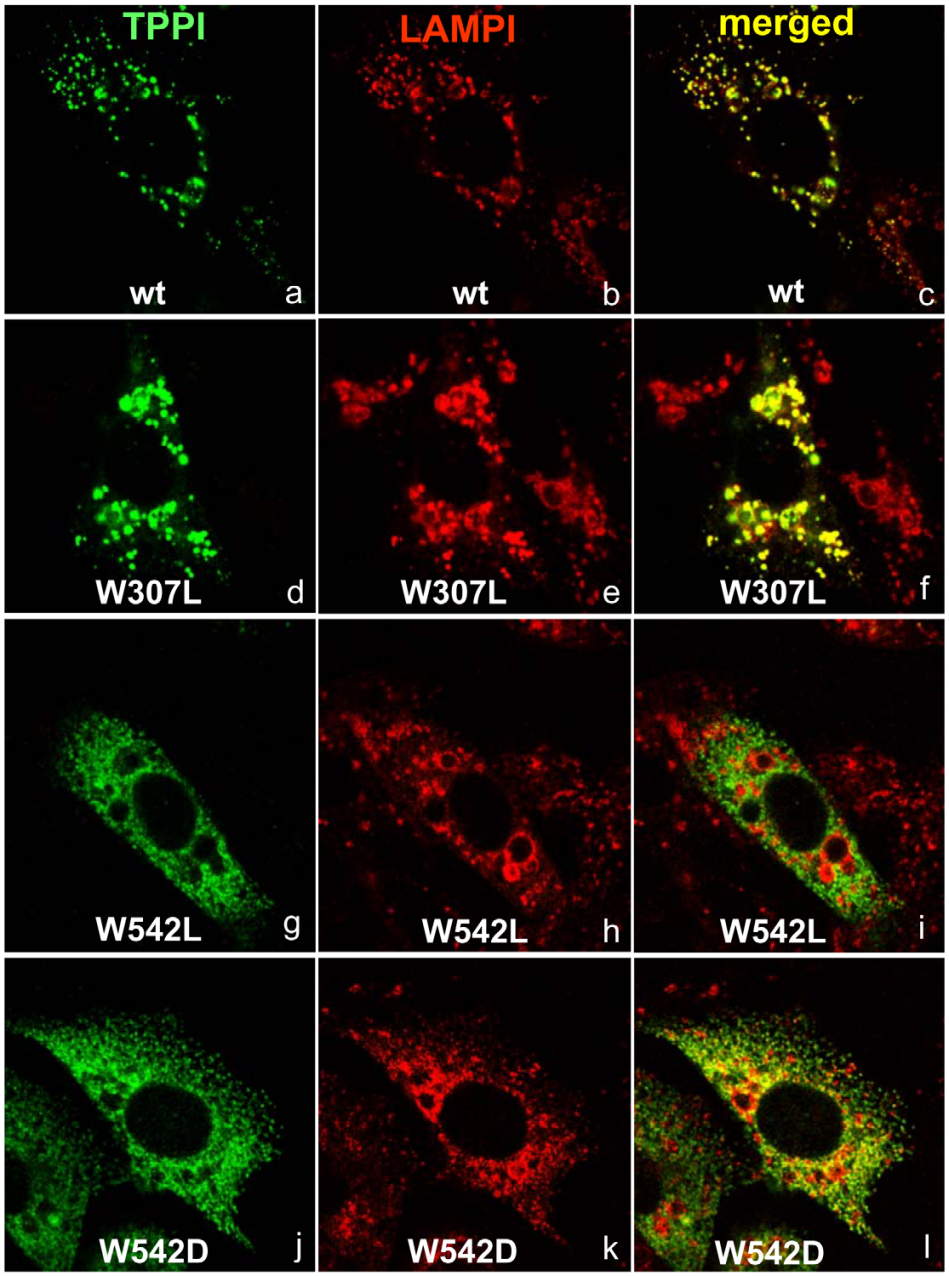
Localization of wt and mutant TPPI in CHO cells. Freshly plated CHO cells at 70% confluency were transiently transfected with mutant plasmids using FugeneHD reagent. After 24 h, cells were immunostained for TPPI (RAS307, rabbit polyclonal) and LAMP (mouse monoclonal). Secondary antibodies labeled with Alexa Fluor 488 (green fluorescence visualizing TPPI) and Alexa Fluor 555 (red fluorescence visualizing LAMP) were used. Upon merging, the yellow color indicates overlap of expressed proteins. Digital images were taken at a magnification of ×1000.

TPPI is a lysosomal peptidase, which is transported by mannose-6-phosphate receptor–mediated organelle targeting into lysosomes [43], [44]. Critical glycosyl residues are added, TPPI is folded into its native conformation in the ER, and the final maturation of pro-TPPI to functionally active TPPI occurs in the lysosomes. Misfolded proteins that were destined for lysosomes accumulate in the ER, and these are rapidly degraded by the cellular quality control machinery [45]. We have evaluated whether the W mutants are processed completely and transported to their functional destination, by using confocal microscopy with organelle specific antibodies (Figure 3 and 4). In transiently transfected CHO cells, double immunostaining for TPPI and LAMP, both of which are lysosomal proteins, and calreticulin, which is a protein that resides in ER, revealed that the W542L mutant is retained in the ER and is only partially transported out to the lysosomes, indicating a lack of complete maturation. This result suggests that W542L mutant may be improperly folded, and as a result does not leave the ER efficiently. Another mutant, W542D, was also partially retained in the ER and transported to the lysosomes indicating a slower processing. While W542L mutant was completely devoid of enzyme activity, modifying the W542 residue with other amino acid residues partially recovered the TPPI activity. The mutant W542D resulted in partial transport into lysosomes, corroborating the measurement of partial activity. Mutation of other W residues had no effect on the processing and targeting into lysosome, as shown by W307L mutant.

### Importance of W residues in autocatalysis of pro-TPPI

**Figure 5:**
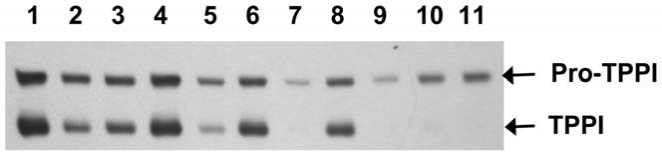
Autocatalytic processing of pro-TPPI. Western blot analysis of the expression and processing of wt and various mutant TPPI cDNAs in transiently transfected CHO cells. CHO cells at confluency were transiently transfected with various plasmids using the FugeneHD reagent. After 48 h, cells were homogenized in sample buffer, and total cellular proteins (25 µg) were resolved on 10% SDS-PAGE. After transferring onto nitrocellulose membrane, TPPI was visualized by chemiluminesce using mouse monoclonal antibody (8C4). Lane 1, wt TPPI; lane 2, mutant W290L; lane 3, mutant W307L; lane 4, mutant W366L; lane 5, mutant W460L; lane 6, mutant W470L; lane 7, mutant W542L; lane 8, mutant W548L; lane 9, mutant W542D; lane 10, mutant W542H and lane 11, mutant W542K.

To further confirm whether the W residues play any critical role in the autocatalytic maturation of the pro-TPPI, we have studied the processing of each of the mutants in CHO cells by Western blot analysis using the 8C4 mouse monoclonal antibody against TPPI [46]. Immunoblots revealed the presence of W542L and W542D mutants as pro-TPPI (Figure 5), indicating a lack of efficient processing to the active enzyme. Two other mutants, namely, W290L and W307L were also found to have reduced autocatalysis as indicated by the presence of the majority of expressed protein as immunoreactive pro-TPPI.

The results obtained by confocal microscopy, Western blot and biochemical assay suggest that the W542 is critical for maintaining the proper tertiary structure of the TPPI. Furthermore, these results also suggest the importance of W542 in the maturation of the precursor, pro-TPPI, to functionally active TPPI. This creates a dilemma as to whether the inhibition seen is due to actually W being modified or due to the protein being misfolded and retained in ER. Our efforts to create truncated TPPI expressing only the mature enzyme were unsuccessful, probably due to improper folding, lack of proper glycosylation or some unknown reason. We have also made unsuccessful attempts at expressing functionally active TPPI fused to secretory fragment of yeast.

### Importance of Ca^2+^ in the autocatalysis of pro-TPPI

**Figure 6:**
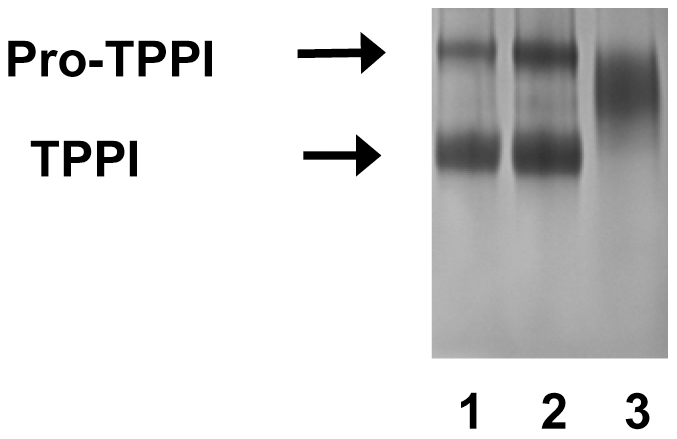
Autocatalytic processing and inhibition of pro-TPPI by EDTA and NBS. Pro-TPPI was incubated with either 10 mM Tris.HCl, pH 7.2 (lane 1); 10 mM EDTA, pH 7.2 (lane 2) or 25 µM NBS (lane 3) in 10 mM Tris.HCl buffer, pH 7.2, at 37°C for 15 min. Thereafter, the pH was reduced to 3.5 by adding ammonium formate, and incubation continued for 5 min. The incubation mixtures were resolved by 10% SDS-PAGE, and proteins were visualized by silver staining.

Recent X-ray crystallographic data revealed the presence of Ca^2+^ in TPPI, the significance of which is currently unclear. An earlier study from our laboratory showed a lack of inhibition of TPPI by EDTA [37]. The W542 that was found to be critical for TPPI activity and pro-TPPI processing is located within the cleft that binds Ca^2+^. To study the importance of Ca^2+^ in TPPI, we have evaluated EDTA and EGTA effects on the processing of pro-TPPI as well as TPPI activity. Both EDTA and EGTA inhibit the processing of pro-TPPI in a concentration-dependent manner. For these studies, pro-TPPI (pH 7.4) was preincubated for 15 min with different concentrations of either chelator (pH 7.4). At this pH, the autocatalytic processing of pro-TPPI is not active, thus allowing the Ca^2+^ chelating to proceed. Thereafter, the pH was lowered to 3.5, and incubation continued for an additional 5 min to allow autocatalysis. At pH 3.5, pro-TPPI is rapidly activated to TPPI, and the majority of enzyme will be in the active form. Ultimately, the TPPI activity was measured by the addition of the tetrapeptide substrate as described in “Experimental Procedures”. About 50% of pro-TPPI processing is lost by incubation with 12 mM EDTA (Table 5 and Figure 6). EDTA was more effective in inhibiting the autocatalysis of pro-TPPI when compared to an equimolar EGTA (data not shown). A preincubation with either chelating agent was found to be necessary to achieve inhibition, which suggests that the Ca^2+^ is tightly bound to TPPI. In contrast, the purified TPPI activity is not inhibited by either, EDTA or EGTA at concentrations up to 25 mM, in line with our earlier observation. These results suggest that Ca^2+^ is important for the proteolytic processing of pro-TPPI but is not essential for the tripeptidyl peptidase activity.

**Table 5:** Inhibition of autocatalytic processing of pro-TPPI by EDTA.

EDTA (mM)	TPPI activity (%)
0	100
3.125	94
6.25	82
12.5	54
25	25

Pro-TPPI was preincubated with indicated EDTA concentration (pH 7.0) for 15 min at 37°C. The reaction mixture was lowered to pH 3.5, and incubation continued for 5 min at 37°C to allow autocatalysis. TPPI activity was initiated by adding the tetrapeptide substrate and measuring the product formed as described in Experimental Procedures.

### NBS inhibits autocatalysis of pro-TPPI

NBS is known to break peptide bonds in proteins [47]. We have evaluated whether the inhibition seen in our studies is the result of proteolysis. Preincubation of the pro-TPPI with NBS completely prevented functional autocatalytic activation to TPPI (Figure 6). In this incubation, the pro-TPPI was processed to a diffuse band with an apparent ∼67-kDa protein. No protein band was observed at the ∼48 kDa area corresponding to the active TPPI, indicating that NBS also prevents the autocatalytic activation of the proenzyme. As expected, no TPPI activity was observed when pro-TPPI was allowed to undergo *in vitro* autocatalysis in the presence of NBS, followed by activity measurement, as described in “Experimental Procedures” (data not shown). The separation by SDS-PAGE of pro-TPPI, following incubation with NBS, EGTA or Ca^2+^, failed to reveal any smaller fragments. This observation suggests that the inhibition of TPPI by NBS is not through peptide bond breakage.

## Discussion

**Figure 7:**
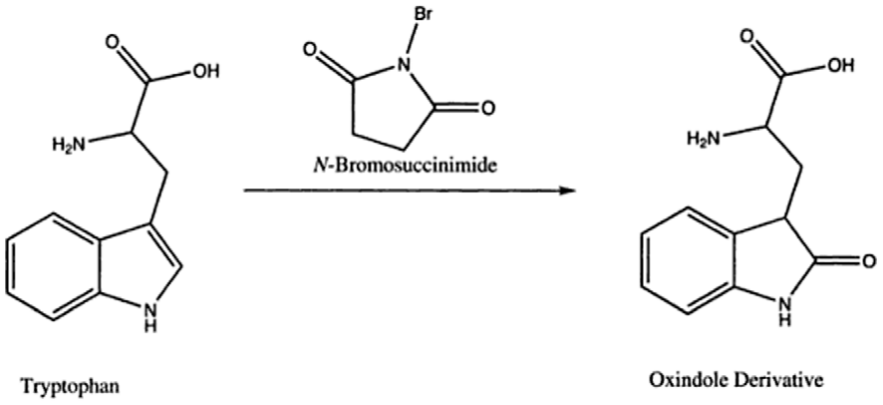
Chemical reaction of NBS with W. NBS reacts with W causing oxidation of the pyrrole part of the indole ring, which results in pyrrole hydrogen pulled towards the ring more tightly.

This report identifies a critical residue in the TPPI sequence that is outside of the active-site triad and is still essential for the enzyme activity. The results presented have demonstrated that W542 plays a pivotal role in the catalysis of TPPI. W residues generally participate in hydrogen bonding through the pyrrole nitrogen, and thus are critical for maintaining the tertiary structure in proteins. Although the pyrrole nitrogen is a very weak base, the hydrogen bonds it forms with other side chain reactive groups contribute significantly to stabilize structures [48]. By performing the reaction of NBS with TPPI in formate buffer at acidic pH, NBS will preferentially oxidize the indole side chain of W residues. The 2,3 double bond of the pyrrole ring of tryptophan, which is relative to the carboxamido group of tryptophan residues bound in peptides, participates in a displacement reaction with NBS (Figure 7). While NBS has been shown to act in several different ways to inhibit the activity of protein molecules, including the breakage of peptide bonds, the present experiments failed to reveal any smaller species from either TPPI or pro-TPPI upon SDS-PAGE, suggesting that the inhibition of activity seen is not due to the breakage of peptide bonds. Thus, the most likely route of NBS inactivation of TPPI is by oxidation of the indole group of a specific W residue. Using the site-directed mutagenesis approach and modifying each of the seven remaining W residues with L, we have identified W542 to be the most likely residue necessary for the TPPI activity.

Recent X-ray crystallographic structures of TPPI have shown presence of octahedrally coordinated Ca^2+^ in the structure [32], [33]. The function of Ca^2+^ was so far unclear. An inspection of the crystal structure of TPPI reveals that W542 is in a region that is involved in octahedrally coordinated binding of the Ca^2+^. The residues involved in binding Ca^2+^ ions are D517, D543, V518, G539, and G541. The critical W542 residue is located between two of these residues G541 and D543, which are involved in this ionic interaction the former providing the main chain carbonyl group and the latter through the carboxyl side chain. Our results have shown that Ca^2+^ is critical for autocatalytic cleavage of pro-TPPI to mature enzyme. This conclusion is deduced from the inhibition of pro-TPPI processing by two Ca^2+^-chelating agents, EDTA and EGTA. These studies identify the importance of maintenance of proper tertiary structure around the tight cleft that binds octahedrally coordinated Ca^2+^. Thus, disruption of this tertiary structure around the Ca^2+^-binding area is detrimental both for the autocatalytic maturation and for the activity of TPPI. All the Ca^2+^ binding residues along with the W542 are conserved across all mammalian TPPI, indicating their crucial importance in maintaining the structure.

**Figure 8:**
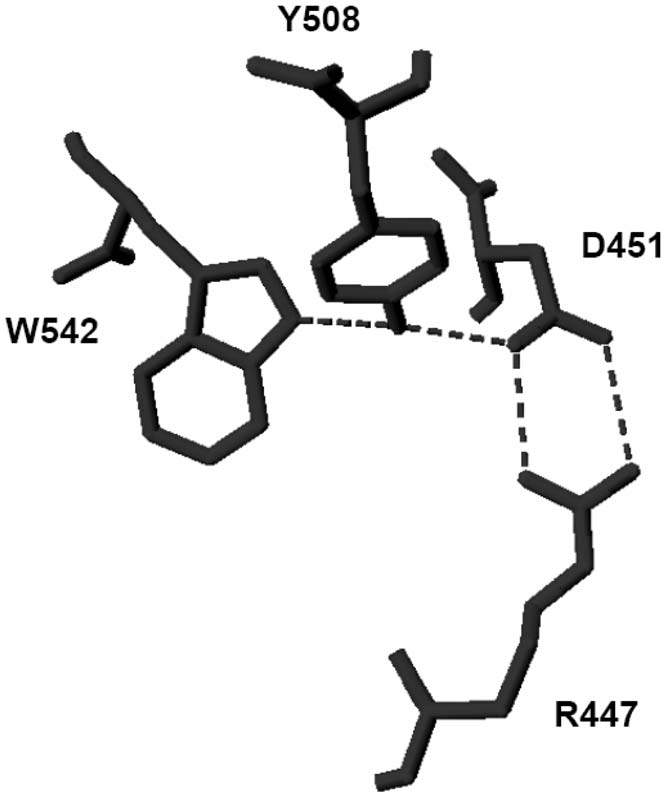
Hydrogen bonding between TPPI residues. Interaction of residues W542, Y508, D451, and R447 through hydrogen bonding. X-ray crystallographic structure of TPPI, 3EDY, was modeled using the software DeepView v 4.01 with the hydrogen-bonding function activated. The hydrogen bridge formed by four amino acids, W542, Y508, D451, and R447, is shown by the dotted lines without the peptide backbone.

**Figure 9:**
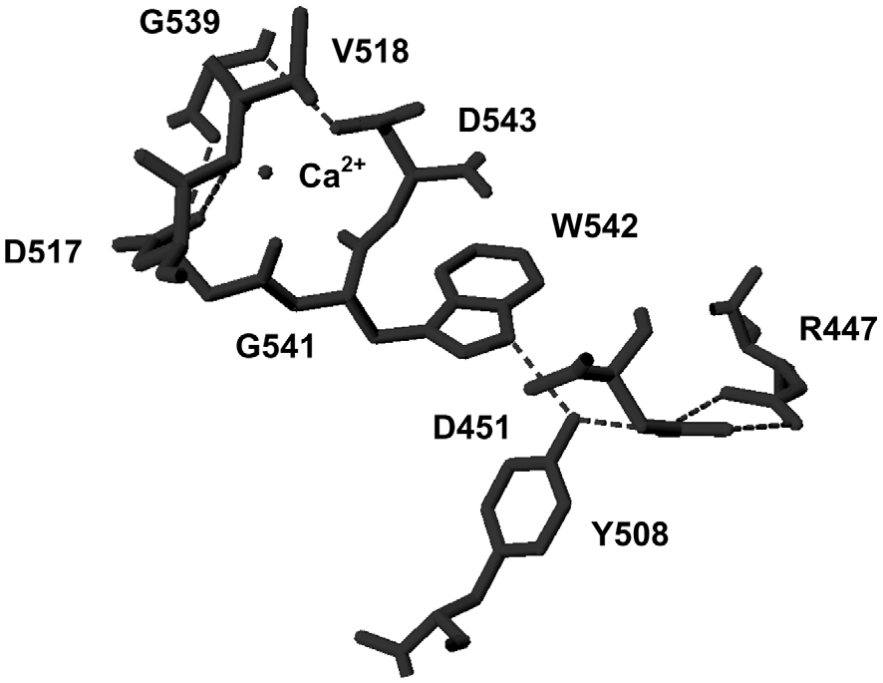
Interaction between Ca^2+^-binding amino acid residues and hydrogen-bonding quartet involving W542. Interaction of residues W542, Y508, D451, and R447 through hydrogen bonding along with the Ca^2+^ binding residues.

We have examined the two X-ray crystallographic structures (3EDY and 3EE6) deposited in the Protein Data Bank using the software DeepView (http://spdbv.vital-it.ch/). Modeling has revealed hydrogen bonding between the W542, Y508, D451 and R447 amino acid residues (Figure 8). These bonds were determined with a minimum and maximum distance of 1.2–2.7 Å respectively. The pyrrole nitrogen in W542 is involved in hydrogen bonding with the hydroxyl group of Y508, which is involved in hydrogen bonding with the carboxylate side chain of D451, which in turn is involved in hydrogen bonding with the guanidinium group of R447 (Figure 8). The NBS reaction with W542 oxidizes the indole nitrogen, thereby precluding it from forming hydrogen bonding between this quartet. This reaction also probably distorts the Ca^2+^ binding by D517, V518, G539, G541 and D543 (Figure 9). Another possibility is that the L substitution totally prevents the Ca^2+^ binding structure, suggesting misfolding and resultant entrapment in the ER. Hence, disruption of the electrophilic nitrogen residue in the W either with NBS or by site-directed mutagenesis resulted in the complete loss of TPPI activity, suggesting that the hydrogen bonding in which it is involved with D451 and Y508 is essential for maintaining the proper conformation of TPPI. This contention is corroborated by the fact that replacing W with other amino acid residues that bear charged side chains instead of neutral L restores partial TPPI activity. It may not be surprising to find this mutation in individuals with LINCL involving W542, Y508 or D451. To date, however, no known mutations have been reported to involve these residues. These three residues are conserved across all mammalian TPPI, suggesting that they contribute significantly to maintaining the proper tertiary structure.

Additionally, the D451 residue that forms a hydrogen bond bridge with Y508 and W542 is localized close to D543, which is involved in octahedrally binding Ca^2+^ ions. Thus, interruption of this hydrophobic pocket, either by NBS that is reacting with W542, or by mutation of R447H, results in the complete loss of TPPI activity. Pal and colleagues, in the x-ray crystallographic data they obtained with TPPI, have shown the molecular basis of one of the mutations, R447H, in an LINCL patient, that results in disruption of a hydrogen bond at D451 [32]. The residues involved in the Ca^2+^ pocket are conserved in the S53 family of serine peptidases, suggesting a crucial role in catalysis. Our data show that Ca^2+^ is important for the autocatalysis rather than the peptidase activity. The carboxylate side chain of D451 is involved in hydrogen bonding with Y508 and R447, of which the Y508 appears to be critical.

An examination of the NCL mutational database for LINCL has shown only one mutation involving W residues in the TPPI (http://www.ucl.ac.uk/ncl). This mutation, however, results in a missense mutation, W460X. Our results with site-directed mutagenesis revealed that mutation of W460 has no effect on the TPPI activity. The presence of a nonsense mutation for W460 in an LINCL patient can be explained by premature termination and a truncated protein devoid of activity, rather than a direct involvement of this W mutant in any critical function in the TPPI structure.

The involvement of Ca^2+^ in the autocatalytic activation of pro-TPPI to TPPI is not unique. Studies with maturation of the *trans-Golgi* network protease, furin, have shown that autocatalysis of this protease is Ca^2+^-dependent and that it does not occur under reducing conditions [49]. The precursor pro-furin, in this instance, is also retained in the ER, similar to what we have observed with the pro-TPPI mutant W542L. The enzyme prohormone convertase 1 (PC1, or SPC3) is another example that undergoes Ca^2+^-dependent autocatalysis [50]. Both furin and PC1 are examples of eukaryotic subtilisin-like serine proteinases. Also, in the prokaryotic subtilisin, the Ca^2+^ is involved in stabilizing the protein structure; however, the enzyme activity is insensitive to chelators [51]. TPPI is also classified as a subtilisin-like serine proteinase [9].

Whether the active-site triad of TPPI comprising S475-E272-D360 residues also participates in the autocatalysis is unclear. The involvement of Ca^2+^ in the processing of the precursor, but not in the actual peptidase activity, suggests that in pro-TPPI, different residues may be involved that cleave off the pro-segment. Additional support for this contention comes from earlier reports of correct autocatalytic processing of a S475L mutant found in an LINCL patient [27], [52]. In patients exhibiting this mutation, the pro-TPPI is processed to the 48k-Da protein; however, the tripeptidyl peptidase activity is absent, suggesting that the active-site residues do not participate in the maturation of TPPI. The function of Ca^2+^ in the pro-TPPI could be two-fold: to stabilize the protein in proper conformation to be able to deliver to its final destination, the lysosome, and to regulate the autocatalytic activity. Once in the lysosome, the acidic pH activates the autocatalytic activity, thereby releasing the active TPPI. After the pro-enzyme activates, the calcium is no longer essential for the peptidase activity. However, it may still be stabilizing the structure, as Ca^2+^ is not easily dissociated. Disruption of the tertiary structure by destroying the hydrogen bonding in the pocket holding the Ca^2+^ will result in the loss of TPPI activity.

Of all the inhibitors tested for TPPI, only a substrate-based derivative of chloromethyl ketone was found to be a potent inhibitor that caused non-competitive inhibition [53]. Chloromethyl ketone derivatives inhibit enzymes that have a serine residue as one of the active-site residues. However, unlike other serine proteinases, the effect of this inhibitor on TPPI was found to be reversible. The other inhibitor that had any effect on TPPI activity was DFP, which also reacts with the active site S475 and inactivates the enzyme. NBS is the third compound that significantly inhibits the TPPI activity without affecting the active site. Studies with such compounds will prove valuable in delineating the critical residues in TPPI catalysis and its structure-function analysis.

## Materials and Methods

### Materials

The QuickChange II site-directed mutagenesis kit was procured from Stratagene, La Jolla, CA. The tetra-peptide substrate GFFL-aminotrifluoromethyl coumarin (GFFL-AFC) was chemically synthesized by Enzyme Systems Products, Livermore, CA. A Qiagen midi kit (Qiagen, Valencia, CA) was used to purify plasmids from bacterial cultures for transfection experiments. All other chemicals were purchased from Sigma Chemical Co, St. Louis, MO. N-Bromosuccinimide (NBS) was recrystallized from water prior to use.

### Methods

#### Site-directed mutagenesis and transformation of competent cells

A wild-type (wt) human TPPI cDNA inserted between the *Kpn*I*/Not*I sites of pcDNA3.1 Hyro was used to introduce site-directed mutagenesis. The construction of the human wt TPPI cDNA has been described earlier [29]. This cDNA contains the H175R mutation, and additionally has another polymorphism that retains A348. Each of the seven W residues in the mature TPPI was altered to L residue according to the instructions supplied by the manufacturer using the primer pairs described in Table 2. In this kit, a complete plasmid strand is first synthesized that incorporates the desired mutation by regular denaturing, annealing and extension cycles, followed by digestion of the template strand and transformation of the mutant plasmid into competent cells.

The mutated plasmids were transformed into HB101 competent cells (Promega, Madison, WI) as per the manufacturer's instructions. Between 4 and 6 colonies were selected, and plasmid DNA was prepared from each bacterial culture by using the Wizard Plus plasmid DNA purification kit (Promega, Madison, WI), following the manufacturer's suggested protocol.

#### DNA sequencing

The insertion of WL mutation was verified by direct sequencing in both the forward and reverse directions. The following sequencing primers were used: Forward: 769F 5′-CACATCAGGCATCAGTAG-3′ and 1206F 5′-GGAACCTTTCCTCATCAC-3′, and reverse: 632R 5′-ATCGCTTACGGATCACAG-3′ and 1300R 5′-GCGCAGGAACTTCGTTAC-3′. Dye terminator cycle sequencing was done in a Beckman Coulter CEQ 8000 sequencer equipped with capillary electrophoresis system according to manufacturer's suggested procedure. Multiple DNA sequence comparisons were done with publicly available ClustalW2 software (http://www.ebi.ac.uk/Tools/clustalw2/index.html) against the NCBI human TPPI mRNA sequence (locus NM_000391).

#### CHO cell culture and transient transfection

CHO cells (ATCC CCL-61) were maintained in F-12 medium supplemented with 10% fetal calf serum, 2 mM glutamine, and antibiotics (penicillin and streptomycin) at 37°C in a humidified atmosphere with 5% CO_2_. One day before transfection, the CHO cells were trypsinized, detached and seeded on 35-mm culture dishes. Cells were transfected by using FuGENE reagent, according to the manufacturer's recommendation, with circular plasmids. Forty eight hours after transfection, cells were trypsinized and collected by centrifugation at 800× g and rinsed once with normal saline.

#### TPPI purification

The ∼48 kDa mammalian TPPI was purified to apparent homogeneity from bovine brain as described earlier [37]. Purified fractions were stored at −20°C in 50 mM ammonium formate buffer, pH 3.5, until further use without any noticeable loss in peptidase activity over extended periods. No proteolytic fragments were apparent upon SDS-PAGE separation followed by silver staining.

#### Pro-TPPI activation

All additions to enzyme preparations were performed on ice, whereas incubations were conducted at 37°C unless otherwise mentioned. Human pro-TPPI was purified from serum-free secretions of CHO-DHFR^–^ cells (ATCC CRL-9096) stably transfected with plasmid encoding the full-length human wt TPPI. Vector preparation, cell transfection, selection and purification procedures were performed essentially as described previously [29]. Purified pro-TPPI was stored frozen in 10 mM Tris-HCl, pH 7.4, containing 1 mM EDTA and 0.2 mM PMSF at −20°C indefinitely without measurable loss in enzyme activity.

At the time of activation, the pro-TPPI was incubated with 25 µM NBS, 10 mM CaCl_2_ or 10 mM EGTA for 15 min. Following this preincubation, the pro-TPPI was activated by autocatalysis in 50 mM ammonium formate buffer, pH 3.5 for 5 min at 37°C, and finally TPPI activity was measured by adding the substrate GFFL-AFC.

#### TPPI activity measurement

TPPI enzyme activity was measured by the sensitive and specific method we developed earlier with slight modifications [12]. Assays were done in 50 mM ammonium formate buffer, pH 3.5, for 5 min at 37°C. The substrate GFFL-AFC, dissolved in 40% dimethylformamide, was used at a concentration of 180 µM. The reaction was terminated by adding 100 µl acetonitrile and an aliquot was directly analyzed by reversed-phase HPLC (HP 1050) on a Microsorb-MV C18 column (4.6 mm ×10 cm, 3 µm, pore size 100A, Varian Inc, CA). Eluents were detected by a variable wavelength ultraviolet (UV) detector at 340 nm, and peaks were integrated by using a HP3395 integrator.

NBS solutions were freshly prepared in distilled water prior to the experiment. EDTA and EGTA solutions were adjusted to pH 7.4 by titration with dilute NaOH. When chemicals were tested for activation or inhibition, they were pre-incubated at 37°C at the final indicated concentrations with the TPPI for 5–15 min prior to the addition of the substrate. Thereafter substrate was added and activity was measured for 5 min at 37°C.

#### SDS-PAGE and Western Blotting

Samples were solubilized in the 2X reducing sample buffer, boiled for 5 min, and loaded onto 10% Tris/HEPES/SDS-polyacrylamide gels (Life Gels). Electrophoretically separated proteins were stained with silver nitrate or electrotransferred onto Optitran BA-S83 reinforced nitrocellulose membrane (Whatman). Nitrocellulose membranes were blocked with 5% nonfat dry milk in PBST, incubated overnight with monoclonal antibody 8C4, an anti-TPP I monoclonal antibody characterized previously [46], then incubated with horse raddish peroxidase–conjugated secondary antibodies diluted 1∶2,000, and developed by using the SuperSignal West Pico chemiluminescent substrate (Thermo Scientific, Rockford, IL). Images were captured either on an X-ray film or by using a CCD camera attached to a Bioimager (UVP system, Upland, CA).

To measure autocatalysis, the pro-TPPI was pre-incubated for 15 min at pH 7.4 with NBS, Ca^2+^ or EDTA. Following the preincubation, autocatalysis was initiated by adding ammonium formate buffer to pH 3.5 and incubating for 5 min. The incubation mixture was mixed with equal volume of 2X SDS-PAGE sample buffer and proteins resolved by 10% SDS-PAGE. After separation, the proteins were fixed using trichloroacetic acid, and visualized by silver staining.

#### Immunofluorescence Microscopy

For double immunostaining, CHO cells grown in LabTek chamber slides (Nunc Nalage International, Rochester, NY) were fixed with methanol for 20 min at 4°C. Nonspecific binding sites were blocked with 10% FCS in PBS for 1 h. After incubation with primary antibodies diluted in 10% FCS in PBS overnight at 4°C, cells were washed with PBS and incubated for 1 h at room temperature with species-specific secondary antibodies conjugated with fluorescent dyes: Alexa Fluor 488 (for TPP I; green fluorescence) and Alexa Fluor 555 (for LAMP I and calreticulin; red fluorescence). The cover slips were mounted with Vectashield medium and viewed with a Nikon Eclipse E600 laser scanning confocal microscope. Omission of the primary antibodies was used as a control of the method.

#### Protein measurements

Protein concentration in the cell extracts was determined following the method of Lowry *et al* [54] using bovine serum albumin as the standard.

#### Molecular modeling

Molecular modeling studies were performed on the x-ray crystallographic structures 1EE6 [32] and 3EDY [33] accessible at the RCSB protein data bank (http://www.pdb.org), using the publicly available software DeepView v 4.0 (http://spdbv.vital-it.ch/).

#### Statistical analyses

Enzyme assays were run in triplicate, and means ± SD were determined for each point.
